# Overlapping and specific bacterial communities in the gut and reproductive system of *Bactrocera dorsalis* (Hendel) (Diptera: Tephritidae) adults

**DOI:** 10.3389/fmicb.2025.1567154

**Published:** 2025-06-02

**Authors:** Wei-Jun Li, Kai-Ping Hu, Xin Zhong, Shui-Lin Song, Cui-Kang Xu, Qing-Xiu Xie, Xiao-Zhen Li

**Affiliations:** Department of Plant Protection, College of Agronomy, Jiangxi Agricultural University, Nanchang, China

**Keywords:** *Bactrocera dorsalis*, gut, reproductive system, bacterial diversity, specific bacterial communities, function annotation

## Abstract

**Background:**

Different insect tissues represent heterogeneous niches with distinct physiological and biochemical characteristics, and therefore host different bacterial communities.

**Methods:**

In this study, those overlapping and specific bacterial communities in the female gut (fG), male gut (mG), female reproductive system (fR), and male reproductive system (mR) of *Bactrocera dorsalis* (Hendel) adults were determined by high-throughput sequencing targeting 16S rRNA gene.

**Results:**

The richness of bacterial taxa based on OTU was higher in fR compared to the other three tissues. Among the 29 identified bacterial phyla, Pseudomonadota, Bacillota, and Bacteroidota were predominant, while among the 48 identified genera, *Enterobacter*, *Kluyvera*, *Asticcacaulis*, *Mesorhizobium*, and *Serratia* were common in the four tissues. fG harbored specific bacterial genus *Morganella*, mG harbored specific bacterial genera *Vagococcus*, *Lactobacillus*, *Lactococcus*, *Lactobacillales*, and *Bacilli*, fR harbored specific bacterial genera *Blastomonas*, *Ralstonia* and *Providencia*, and mR harbored specific bacterial genera *Sphingobacteriia*, *Asticcacaulis*, *Caulobacter*, *Caulobacterales*, *Bradyrhizobium*, and *Luteimonas*. In the 35 annotated KEGG pathways, high-abundance bacterial taxa were mainly enriched in these pathways of membrane transport, carbohydrate metabolism, amino acid metabolism, replication and repair, and energy metabolism, while low-abundance bacterial taxa were involved in these pathways of cardiovascular diseases, circulatory system, and excretory system. The abundances of the 5 pathways associated with cardiovascular diseases, circulatory system, excretory system, membrane transport, and polysaccharide biosynthesis and metabolism exhibited greater variations among fG, mG, fR, and mR. Among them, the two pathways abundances of cardiovascular disease and circulatory system were higher in the reproductive system, whereas the other three pathways abundances were higher in the female gut.

**Conclusion:**

Our study revealed the abundance, composition and function of overlapping and specific bacterial communities in the gut and reproductive system of *B. dorsalis*, providing valuable information for inhibiting the occurrence of *B. dorsalis* by interfering with these functional bacterial communities in tissues.

## 1 Introduction

Insects often coexist with symbiotic bacteria living inside their bodies (Amdam, 2012; Shi W. et al., 2012). Of special interest are insect-associated bacterial communities, some being ubiquitous across different insect tissues, others being specific to particular tissues (de Bekker et al., 2013; Santos et al., 2023). For example, *Enterobacter*, *Kluyvera*, and *Asticcacaulis* are very common in the gut and reproductive system of insects, such as *Phlebotomus papatasi* (Scopoli) (Maleki-Ravasan et al., 2014), *Ceratitis capitata* (Wiedemann) (Ben-Yosef et al., 2008), and *Bactrocera cacuminata* (Hering) (Thaochan et al., 2010). In contrast, *Wolbachia* strains *w*Cer1 and *w*Cer2 are gram-negative bacteria that exhibit significant tissue specificity, primarily residing in the reproductive system of insects (Riegler and Stauffer, 2002). Insect-associated bacteria may establish a mutualistic symbiotic relationship with insects (Adair and Douglas, 2017), where insects provide space and nutrient for their associated bacteria, while the bacteria support insect growth and health by aiding insects in obtaining nutrients or resisting pathogens (Kandasamy et al., 2022). An example of such a mutualistic relationship is that two lactic acid-producing bacteria, *Enterococcus casseliflavus* and *Lactococcus lactis*, may increase the resistance of *Bactrocera dorsalis* (Hendel) to β-cypermethrin (Zeng et al., 2024).

Different insect tissues exhibit physiological and functional differences. For example, insect guts may contain specific substances, such as digestive enzymes, proteins, and vitamins, which aid in the degradation and absorption of dietary ingredients (Douglas, 2009); and insect reproductive systems contain nutrients and enzymes related to the formation and maturation of eggs or sperms, helping insects to reproduce offspring (Gavriel et al., 2011). The different tissues of insects represent heterogeneous niches with distinct physiological characteristics, and may thus host their own specific bacterial communities (Santos et al., 2023). This heterogeneity may drive the adaptive evolutionary and tissue-specific distribution of insect-associated bacterial communities. Meanwhile, insect-associated bacteria may also require localization in specific insect tissues to function optimally (Feldhaar and Gross, 2009; Lu et al., 2016).

Bacterial communities in different insect tissues have received considerable attentions. We retrieved over 50 articles from PubMed database, exploring the diversity of bacteria in the gut of insects, such as *Laodelphax striatellus* (Fallen) (Zhang et al., 2020), *Recilia dorsalis* (Motschulsky) (Huang et al., 2023), *Schistocerca gregaria* Forskål (Dillon et al., 2010), *Sogatella furcifera* (Horvath) (Bing et al., 2020), and *Zeugodacus cucurbitae* (Coquillett) (Lakshmi et al., 2023), and six articles retailing the bacterial communities in the reproductive system of insects, such as *B. dorsalis* (Shi Z. et al., 2012), *B. minax* (Enderlein) (Wang A. L. et al., 2014), and *Blattella germanica* L. (Kakumanu et al., 2018). However, few studies were conducted on those overlapping and unique bacterial communities in specific insect tissues. Here, we only found 2 similar articles: one recording the overlapping bacteria between the gut and feces of *B. germanica* (Kakumanu et al., 2018), and the other one determining the overlapping microbiota between the intestinal and reproductive tract of chickens (Shterzer et al., 2020). Studying those overlapping and specific bacterial communities in various insect tissues may offer insights into the intrinsic relationship between bacterial communities and the function of tissues associated with bacteria.

*Bactrocera dorsalis* (Hendel), also known as the oriental fruit fly, is a highly invasive pest of fruits and vegetables, infesting a wide range of crops including citrus, mango, carambola, and guava (Wang H. et al., 2014; Li X. Z. et al., 2024). Due to difference in physiological and biochemical characteristics, the gut and reproductive system of *B. dorsalis* adults may harbor their specific bacterial communities, in addition to sharing some common bacterial communities (de Bekker et al., 2013). Phylogenetic analyses have shown that the gut of *B. dorsalis* harbors bacterial genera, such as *Enterobacter*, *Citrobacter*, *Klebsiella*, *Pantoea*, *Pectobacterium*, and *Serratia* (Gwokyalya et al., 2024; Yong et al., 2017; Liu et al., 2016; Behar et al., 2008), whereas its reproductive system contains bacterial species, namely *Enterobacter sakazakii*, *Klebsiella oxytoca*, *Klebsiella pneumoniae*, *Raoultella terrigena*, and *Enterobacter amnigenus* (Shi Z. et al., 2012). However, the overlapping and specific bacterial communities, particularly the functional pathway underlying their enrichment, in the gut and reproductive system of *B. dorsalis* still remained an area ripe for further exploration.

This study aimed to elucidate the overlapping and specific bacterial communities present in the gut and reproductive system of male and female *B. dorsalis*. The gut and reproductive system was chosen here because they are the main functional tissues of *B. dorsalis* and the optimal ecological niches for the development and proliferation of bacteria (He et al., 2022; Yin et al., 2024). Due to the differences in physiological functions and nutritional components among different tissues (Douglas, 2009; Gavriel et al., 2011), we hypothesized that there would also be some variations in bacterial taxa, as well as the abundance and functions of bacteria communities within each tissue. Our study would provide a comprehensive catalog of bacterial taxa present in *B. dorsalis* different tissues, contributing to functional study of important bacteria.

## 2 Materials and methods

### 2.1 Sample preparation

The laboratory population of *B. dorsalis* was derived from 50 to 100 infested navel oranges (*Citrus sinensis* Osbeck cv. Newhall) collected from an orchard (115°65′E, 25°97’N) in Ganzhou, Jiangxi Province, China in late October, 2018. Larvae from these infested oranges were isolated, and subsequently fed with an artificial diet consisting of wheat bran (300 g), beer yeast (60 g), white sugar (40 g), potassium sorbate (0.8 g), methyl-p-hydroxybenzoate (0.8 g), and water (1,000 mL) until pupation. Pupae were kept in a plastic tray containing 5 cm of loose wet soil (approximately 400 cm^3^) until adults emerged. Newly emerged adults were separated by sex, and housed separately in an insect-rearing box (78 × 50 × 55 cm), where they were provided with a diet consisting of beer yeast, diluted honey (1%), and water (Li W. J. et al., 2024). During the feeding process, the *B. dorsalis* population was maintained at 26.5 ± 2°C with 75 ± 5% relative humidity under a 14 h: 10 h (light: dark) photoperiod cycle.

Before sampling, 5-day-old virgin flies were starved for 12 h to eliminate resident bacterial communities retained in their guts and reproductive systems (Andongma et al., 2015). Flies were then surface-sterilized with a 75% ethanol solution for 2–3 min, followed by three rinses in sterile water before dissection. These flies were carefully dissected using a blade on a plate containing 3 mL sterile phosphate-buffered saline (PBS, pH 7.4) underneath a stereomicroscope (CNoptec, Chongqing, China). The female gut (fG), male gut (mG), female reproductive system (fR), and male reproductive system (mR), obtained through dissection, were transferred separately into 2 mL centrifuge tubes each containing 1 ml of extract solution [TIANamp Genomic DNA Kit (TIANGEN, Beijing, China)]. These tissues/samples were then homogenized. Each tissue/sample type comprised three biological replicates, with each replicate consisting of 30 guts or reproductive systems. All samples were snap-frozen in liquid nitrogen, and subsequently stored at −80°C until DNA extraction.

### 2.2 DNA extraction and PCR amplification

DNA was extracted from the above-mentioned frozen homogenized samples using the TIANamp Genomic DNA Kit (TIANGEN, Beijing, China), following the manufacturer’s protocol. The integrity of the extracted DNA was verified by 1.5% agarose gel electrophoresis, and its concentration was determined by measuring the absorbance at 260 nm using a Nanodrop spectrophotometer (Thermo Fisher Scientific, Madison, WI, USA). For taxonomical profiling of bacteria, the universal primers, forward 338F (5′-ACTCCTACGGGAGGCAGCAG-3′) and reverse 806R (5′-TACHVGGGTWTCTAAT-3′) (Johnson et al., 2019), were used to amplify the V3–V4 hypervariable regions of the bacterial 16S rDNA gene. Both the forward and reverse primers were tagged with linker sequences, pad and Illumina adapter. These primers were designed to incorporate an 8-nucleotide (nt) barcode sequence to accommodate multiple samples. Polymerase chain reaction (PCR) was performed in a total reaction volume of 12.5 μL per sample. Each 12.5 μL reaction mixture comprised 0.5 μL of extracted DNA template, 0.25 μL of each forward and reverse primer, 5 μL of 2 × Hot Start PCR Master Mix (Invitrogen) and 6.5 μL of ddH_2_O. The PCR cycling conditions were as follows: initial denaturation at 95°C for 5 min, followed by 30 amplification cycles at 94°C for 30 s, annealing 56°C for 30 s, extension at 72°C for 60 s, and a final extension step at 72°C for 10 min.

### 2.3 High-throughput sequencing and bioinformatics analysis

The PCR amplification products were verified by 1.5% agarose gel electrophoresis, purified using the AMPure XT beads kit (Beckman Coulter, Beverly), CA, United States), and quantified using the Library Quantification Kit for Illumina (Kapa, Woburn, MA, United States). The length distribution of the DNA fragments was analyzed using the Agilent 2100 Bioanalyzer (Agilent, Palo Alto, CA, United States). The purified 16S amplicons from each sample was pooled in equimolar amounts and subjected to emulsion PCR to generate amplicon libraries (Andongma et al., 2015). These libraries were paired-end sequenced (PE250) using the Illumina MiSeq sequencer (Illumina, San Diego, CA, United States) on the Illumina MiseqTM 2500 platform at Sangon Biotech (Shanghai, China), following the standard protocols.

After sequencing all samples, the raw paired-end reads of each sample replicate were merged using FLASH v3.3, with a minimum overlap of 10 bp defined to obtain raw tags (Magoč and Salzberg, 2011). Paired-end sequences were then trimmed to remove mismatches in the barcode, more than two primer mismatches, homopolymers shorter than 200 bp or longer than 8 bases, and chimera sequences. These were done using the script Reads_Quality_Length_distribution.pl in Trimmomatic v3.0 (Bolger et al., 2014) and the UCHIME v4.2.4 (Edgar et al., 2011). Quality-filtered and trimmed sequences were analyzed using the quality control process of FastQC v0.11.5 (Brown et al., 2017). Operational Taxonomic Unit (OTU) was generated using an open-reference OTU-picking strategy, based on the Usearch v5.2.0 algorithm with a 97% similarity cutoff level (Edgar, 2010). OTUs with low abundance (with a minimum combined abundance threshold of 50) and OTUs belonging to host DNA were removed. The remaining OTUs were identified using UPARSE software v7.0.1001 (Edgar, 2013), and classified into various taxa at the phylum, class, and genus levels against SILVA database (Release138.1) using the RDP classifier v2.12 (Quast et al., 2013). Full-length 16S rRNA sequences were analyzed using SnapGene v4.3.6, and the resulting contigs for each tissue DNA sample were then compared with a corresponding database from NCBI using BLAST to identify bacterial species (Wang A. L. et al., 2014). We counted the numbers of representative reads for each OTU determined at 3% dissimilarity level at each taxonomic level, and calculated the proportion of each group within samples.

### 2.4 Diversity and function of bacterial community

The OTU abundance statistics table for each sample was obtained by comparing all tags against OTUs using the USEARCH_global (Edgar, 2010). The alpha diversities of bacterial communities were calculated using Mothur v1.31.2 (Schloss et al., 2009), based on both phylogenetic distances and non-phylogenetic metrics, and beta diversities were estimated using QIIME v1.8.0 based on the weighted UniFrac distance metrics (Lozupone and Knight, 2005; Caporaso et al., 2010). Principal co-ordinates analyses (PCoA) were conducted using QIIME v1.8.0 to assess the dissimilarities in bacterial community composition among tissues. The homology relationships of bacterial 16S rRNA gene sequences in the samples, when compared against sequences held in the GenBank database, were identified using the BLAST algorithm. The closest relatives, based on percent homology, were used to report taxonomic affiliations at the phylum, genus, and species levels (Altschul et al., 1990). LEfSe clustering and Linear Discriminant Analysis (LDA) was performed using LEfSe v1.1.0 to identify specific and dominant bacteria communities (Segata et al., 2011). We employed the PICRUSt2 (Phylogenetic Investigation of Communities by Reconstruction of Unobserved States) algorithm to extrapolate the potential function of those bacteria communities from different samples by comparing them with reference sequences in the KEGG database (Langille et al., 2013). During this process, we also assessed the abundance of KEGG pathways by assigning sequences to specific KEGG Orthology (KO) numbers, based on the 16S rRNA gene sequence data of OTUs.

### 2.5 Data statistics

The Chao1, ACE, Shannon, and Simpson indices of bacterial community in various tissues of *B. dorsalis* were calculated using Mothur v1.31.2 (Schloss et al., 2009). The significance of the diversity indices (means ± SE) among tissues were evaluated using SPSS v19.0 (IBM, Armonk, NY, United States) through one-way analysis of variance (ANOVA), followed by Tukey’s honest significant difference (HSD) test (*P* < 0.05). PCoA patterns were visualized using QIIME v1.8.0 (Gu F. et al., 2024), and circular cladogram were created using R package v3.4.1 with default parameters (Segata et al., 2011). Other related graphics were drawn using GraphPad Prism v8.0.1 (Swift, 1997).

## 3 Results

### 3.1 General feature of 16S rRNA amplicon sequence

Bacterial communities in the fG, mG, fR and mR of *B. dorsalis* adults were quantified by high-throughput sequencing targeting 16S rRNA gene. After removing pair-end mismatches, homopolymers, and chimeric sequences, we obtained a total of 363,143 high-quality clean reads, with an average read length of 420.50 bp, spanning the V3–V4 variable regions of the 16S rDNA. These clean reads were distributed among the four types of tissues as follows: fG (11.94%), mG (11.71%), fR (35.62%), and mR (40.73%) (Table 1). All of these sequence data generated have been deposited in the NCBI system^1^ under the accession number: PRJNA1122372.

**TABLE 1:** Statistical analysis of 16S rDNA amplicon sequence.

Samples^a^	Barcode^b^	Raw reads	Mean length of raw reads (nt)	Clean reads	Mean length of clean reads (nt)
fG1	CCTTCT	14,806	460.38	14,469	419.34
fG2	TTGTAG	17,610	466.79	17,269	426.67
fG3	AACTAT	11,932	464.21	11,617	426.12
mG1	AGGCGG	14,699	462.18	14,315	423.10
mG2	TCTATT	12,200	465.13	11,972	425.19
mG3	CTGACG	16,948	459.29	16,249	422.34
fR1	TATCTG	41,265	456.22	39,874	418.50
fR2	GTTGTT	46,625	462.72	45,530	422.91
fR3	CGTGGT	45,424	459.44	43,955	421.03
mR1	ACTGCG	60,636	438.69	53,827	412.44
mR2	TTAATT	49,440	457.38	46,209	417.50
mR3	GTATCT	53,250	438.97	47,857	410.81

^a^Sample was named according to tissue types.

^b^Barcodes represent the label used for distinguishing sequencing samples. fG, female gut; mG, male gut; fR, female reproductive system; mR, male reproductive system.

**FIGURE 1:**
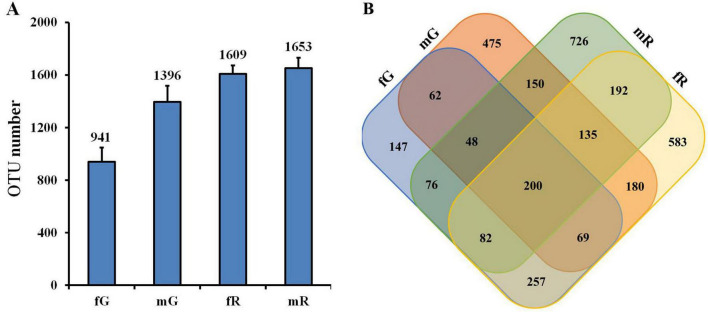
Comparisons of bacterial community based on the OTUs in the fG, mG, fR, and mR of *B. dorsalis*. **(A)** OTU number in each tissue. **(B)** Venn diagram shows those shared and specific OTUs among the four tissues. OTUs were defined based on 3% sequence divergence.

The 363,143 clear reads mentioned-above were binned to 3,382 OTUs at a 97% similarity threshold (Supplementary Table S1). Among them, 941 OTUs were from fG, 1,396 OTUs from mG, 1,609 OTUs from fR, and 1,653 OTUs from mR. Obviously, the number of OTUs in the reproductive system, especially in the male reproductive system, was higher than those in the gut (*F* = 11.77; df = 3, 8; *P* < 0.01) (Figure 1A). Venn diagram showed that the number of specific OTUs in fR (583) and mR (726) was higher than that in fG (147) and mG (475). There were 379 OTUs overlapped between fG and mG, 609 OTUs overlapped between fR and mR, 608 OTUs overlapped between fG and fR, and 533 OTUs overlapped between mG and mR. The four tissues/samples (fG, mG, fR, and mR) had 200 shared OTUs (Figure 1B).

### 3.2 Diversity characteristics of bacterial community

The Chao1, ACE, Shannon, and Simpson indices for the bacterial community in fG, mG, fR, and mR, based on OTUs, were estimated using the phylogenetic distances and non-phylogenetic metrics. Both the Chao1 and ACE indices showed that the richness of the bacterial community in fR was higher than those in fG (Chao1: *n* = 3, *p* = 0.026; ACE: *n* = 3, *p* = 0.048) and mG (Chao1: *n* = 3, *p* = 0.045; ACE: *n* = 3, *p* = 0.024). The Shannon (*n* = 3, *p* = 0.226) and Simpson (*n* = 3, *p* = 0.307) indices suggested that there were no significant differences in the taxonomic diversity of the bacterial community among the fG, mG, fR and mR of *B. dorsalis* (Table 2).

**TABLE 2:** Bacterial diversity indices estimated for the four tissues of *B. dorsalis* adults.

Sample	Chao1	ACE	Shannon	Simpson	Coverage (%)
fG	650.28 ± 30.42 a	858.81 ± 29.45 a	3.02 ± 0.16 a	0.12 ± 0.02 a	98.99
mG	698.24 ± 89.20 a	775.48 ± 36.75 a	3.53 ± 0.37 a	0.09 ± 0.02 a	99.26
fR	1038.73 ± 22.00 b	1180.18 ± 78.21 b	3.74 ± 0.23 a	0.07 ± 0.02 a	98.53
mR	766.04 ± 177.73 ab	771.28 ± 184.30 ab	4.26 ± 0.61 a	0.06 ± 0.03 a	99.56

There are three replicates for each type of tissues. Values are means ± standard error (SE). Different letters (a or b) in the same column denote significant differences on diversity indices among the four tissues of *B. dorsalis*.

**FIGURE 2:**
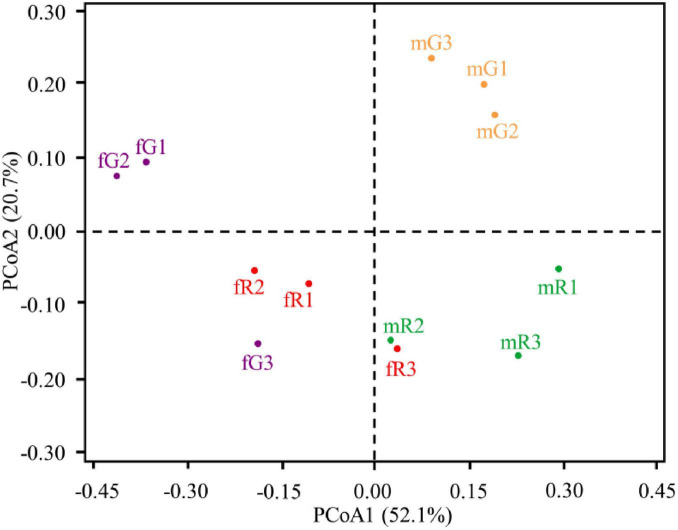
Principal co-ordinates analysis (PCoA) illustrating differences in the taxonomic composition of bacterial communities among independent replicates of four tissue samples (fG, mG, fR, and mR). Dissimilarity in bacterial community composition is based on the weighted UniFrac distance metrics. Percentage of variation explained by each principle component is indicated on axis.

PCoA yielded two main axes, which represented 72.80% of the total variation in bacterial community composition. In this PCoA pattern, we observed a considerable dissimilarity in the composition of bacterial community among fG, mG, fR, and mR, and an obvious clustering of three biological replicates within each specific tissue. By comparison, we also found that the composition of bacterial community between fR and mR was similar, whereas that between fG and mG was distant (Figure 2).

### 3.3 Identification of bacterial community

#### 3.3.1 Bacterial phyla

**FIGURE 3:**
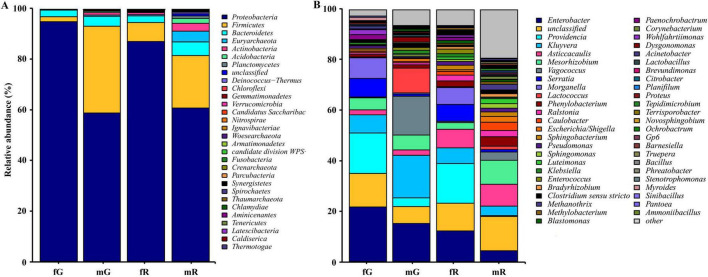
Taxonomic classification of bacterial communities associated with the different tissues of *B. dorsalis* adults. **(A)** Relative abundance of bacteria at phylum level. **(B)** Relative abundance of top 49 bacteria at genus level. The term “other” includes all un-annotated bacterial communities.

We identified a total of 29 bacterial phyla, including *Pseudomonadota*, *Bacillota*, *Bacteroidota*, *Euryarchaeota*, *Actinobacteria*, *Acidobacteria*, *Planctomycetes*, *Deinococcus-Thermus*, *Chloroflexi*, *Gemmatimonadetes*, *Verrucomicrobia*, and others, in addition to some unknown taxa. The three bacterial phyla *Pseudomonadota*, *Bacillota*, and *Bacteroidota* were highly abundant in the fG, mG, fR, and mR of *B. dorsalis*. Among them, *Pseudomonadota* was the most abundant phylum, with relative abundances of 94.99% in fG, 86.85% in fR, 58.69% in mG, and 60.58% in mR. The abundances of *Bacillota* and *Bacteroidota* were relatively high, particularly in mG (*Bacillota*: 34.43%; *Bacteroidota*: 3.91%) and mR (*Bacillota*: 20.34%; *Bacteroidota*: 5.48%). Notably, high abundances of *Euryarchaeota* (4.05%), *Actinobacteria* (3.79%), and *Acidobacteria* (1.92%) were observed only in mR (Figure 3A).

#### 3.3.2 Bacterial genera

We identified 48 genera with relatively high abundance, including *Enterobacter*, *Providencia*, *Kluyvera*, *Asticcacaulis*, *Mesorhizobium*, *Vagococcus*, *Serratia*, *Morganella*, *Lactococcus*, *Phenylobacterium*, *Ralstonia*, *Caulobacter*, *Escherichia*, and *Sphingobacterium*, and others. In fG, the five most abundant genera were *Enterobacter* (21.77%), *Providencia* (16.06%), an unidentified genus (13.71%), *Morganella* (7.91%), and *Serratia* (6.86%). In mG, the five most abundant genera were *Kluyvera* (16.67%), *Vagococcus* (15.82%), *Enterobacter* (14.98%), *Lactococcus* (10.02%), and *Mesorhizobium* (6.22%). In fR, the five most abundant genera were *Providencia* (15.30%), *Enterobacter* (11.83%), an unidentified genus (11.39%), *Asticcacaulis* (7.28%), and *Serratia* (6.96%). In mR, the five most abundant genera were an unidentified genus (13.14%), *Mesorhizobium* (9.04%), *Asticcacaulis* (8.75%), *Enterobacter* (4.26%), and *Phenylobacterium* (2.84%) (Figure 3B). In addition, we did not detect *Novosphingobium*, *Rhizobium*, and *Gaiella* in fG, *Paenochrobactrum*, *Wohlfahrtiimonas*, and *Lampropedia* in mG, *Comamonas*, *Prolixibacter*, and *Tissierella* in fR, and *Gaiella*, *Bdellovibrio*, and *Parasegetibacter* in mR.

#### 3.3.3 Bacterial species

The top 10 bacterial species were *Enterobacter ludwigii*, *Enterococcus faecalis*, *Citrobacter freundii*, *Gibbsiella quercinecans*, *Citrobacter murliniae*, *Klebsiella pneumoniae*, *Lactococcus lactis*, *Citrobacter koseri*, *Gluconobacter oxydans*, and *Stenotrophomonas maltophilia*. Among them, *Citrobacter murliniae* was the most abundant species in fG, with a relative abundance of 0.34%. In mG, *Enterococcus faecalis* was the most abundant species, with a relative abundance of 1.61%. *Enterobacter ludwigii* was the most abundant species in both fR and mR with relative abundances of 2.62 and 4.80%, respectively. Notably, *Gibbsiella quercinecans* was absent in both fG and mG (Table 3).

**TABLE 3:** Abundance of top 10 bacteria species in the fG, mG, fR, and mR of *B. dorsalis.*

Species	Accession	Bestmatch Genbank%	Percentage (%)			
			**fG**	**mG**	**fR**	**mR**
*Enterobacter ludwigii*	NR042349.1	99.72	0.013	0.962	2.615	4.803
*Enterococcus faecalis*	KF179518.1	99.86	0.162	1.610	2.377	0.581
*Citrobacter freundii*	M59291.1	100.00	0.112	1.328	0.051	0.074
*Gibbsiella quercinecans*	NR117526.1	97.54	–	–	0.357	0.007
*Citrobacter murliniae*	NR028688.1	99.72	0.342	0.039	0.171	0.194
*Klebsiella pneumoniae*	NR037084.1	99.86	0.146	0.337	0.002	0.021
*Lactococcus lactis*	M55156.1	99.86	0.004	0.004	0.294	0.142
*Citrobacter koseri*	NR117751.1	99.93	0.211	0.0148	0.003	0.003
*Gluconobacter oxydans*	AB003955.1	98.54	0.002	0.004	0.008	0.174
*Stenotrophomonas maltophilia*	NR113648.1	98.72	0.050	0.008	0.113	0.004
Others	–	–	97.691	95.694	94.010	93.997

#### 3.3.4 Specific and dominant bacterial taxa

**FIGURE 4:**
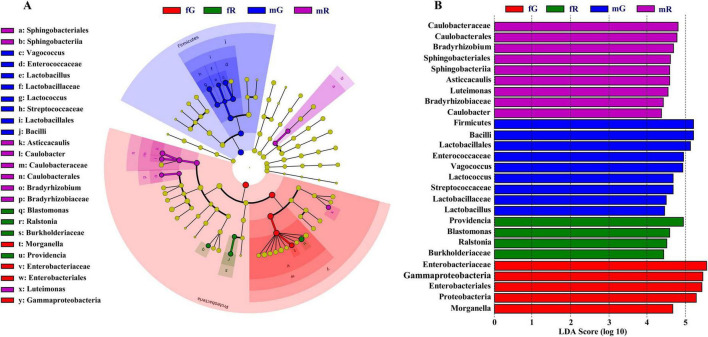
Diagram of specific and dominant bacterial taxa based on LEfSe analysis. **(A)** Circular cladogram starting from the innermost circle: phylum, class, order, family and genus. Each node represents a taxa and the larger the node, the higher the abundance of that taxa. Yellow nodes represent those bacterial taxa that there are no significant differences among different tissues. The other color nodes, such as red nodes, represent those bacterial taxa that there are significant differences among different tissues. Names of specific bacterial taxa are showed in the legend on the left. **(B)** Bar chart created based on the LDA score. Four colors (purple, blue, green and red) in this chart are used to distinguish those bacterial taxa in fG, mG, fR, and mR, respectively. The larger the LDA score, the greater the difference, indicating that the abundance in this bacterial taxon is higher than those in other bacterial taxa.

Specific bacterial taxa in the fG, mG, fR, and mR of *B. dorsalis* were detected using LEfSe analysis. The circular cladogram showed that fG harbored specific bacterial taxa, including the class Gammaproteobacteria, the order *Enterobacteriales*, the family *Enterobacteriaceae*, the genus *Morganella*; mG harbored specific bacterial taxa, including the families *Enterococcaceae*, *Lactobacillaceae*, and *Streptococcaceae*, the genera *Vagococcus*, *Lactobacillus*, *Lactococcus*, *Lactobacillales* and *Bacilli*; fR harbored specific bacterial taxa, including the family Burkholderiaceae, the genera *Blastomonas*, *Ralstonia* and *Providencia*; mR harbored specific bacterial taxa, including the order *Sphingobacteriales*, the families *Caulobacteraceae* and *Bradyrhizobiaceae*, the genera *Sphingobacteriia*, *Asticcacaulis*, *Caulobacter*, *Caulobacterales*, *Bradyrhizobium*, and *Luteimonas* (Figure 4A).

The dominant bacterial taxa in the fG, mG, fR, and mR of *B. dorsalis* were also determined based on the LDA score. The LDA analysis showed that there were 5 bacterial taxa in fG, 9 in mG, 4 in fR, and 9 in mR, each with LDA scores > 4. Among them, there were 3 dominant phyla (*Bacillota*, *Gammaproteobacteria*, and *Pseudomonadota*), 1 dominant class (*Bacilli*), 2 dominant Orders (*Lactobacillales* and *Enterobacteriales*), and 1 dominant family (*Enterobacteriaceae*) in mG or fG, each with LDA scores > 5 (Figure 4B).

### 3.4 Function annotation of bacterial community

**FIGURE 5:**
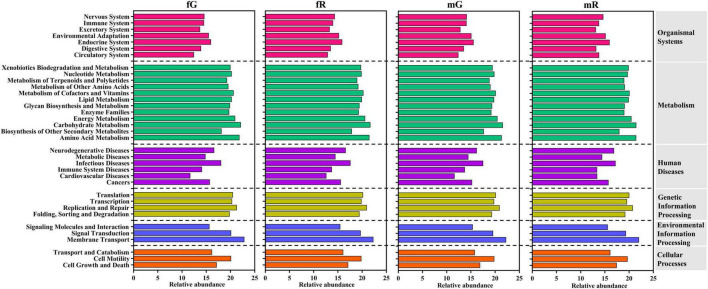
Function classification and annotation of bacterial communities from the fG, mG, fR, and mR of *B. dorsalis* based on KEGG pathway analysis.

KEGG pathway analysis showed that the bacterial communities from the fG, mG, fR, and mR of *B. dorsalis* were annotated as being associated with 6 primary pathways, as organismal systems, metabolism, human diseases, genetic information processing, environmental information processing, and cellular processes. Among them, the metabolism pathway contained 12 subcategories, the organismal systems pathway contained 7 subcategories, while the cellular processes pathway, and environmental information processing pathway each contained only 3 subcategories. In these subcategories pathways, high-abundance bacterial communities (with relative abundance > 20) were enriched in the 5 pathways (subcategories): membrane transport, carbohydrate metabolism, amino acid metabolism, replication and repair, and energy metabolism. Low-abundance bacterial taxa (with relative abundance < 14) were mainly involved in the 3 pathways (subcategories): cardiovascular diseases, circulatory system, and excretory system (Figure 5).

The differences in the relative abundance of 35 pathways (subcategories) enriched by bacterial communities were revealed. The relative abundances of the 5 pathways associated with cardiovascular diseases, circulatory system, excretory system, membrane transport, and glycan biosynthesis and metabolism exhibited greater variations among the four tissues of *B. dorsalis*. Among them, the abundances of the 2 pathways related to cardiovascular diseases and circulatory system were higher in the reproductive system (fR: 12.62 and mR: 13.41 for cardiovascular diseases; fR: 12.91 and mR: 13.79 for circulatory system), compared to the gut (fG: 11.72 and mG: 11.64 for cardiovascular diseases; fG: 12.47 and mG: 12.42 for circulatory system). The abundances of the other 3 pathways were all highest in fG, with values of 13.73 for excretory system, 22.87 for membrane transport, and 19.89 for glycan biosynthesis and metabolism among the four tissues. In addition, the relative abundances of the remained 30 pathways exhibited relatively minor variations among the four tissues of *B. dorsalis* (Figure 5).

**FIGURE 6:**
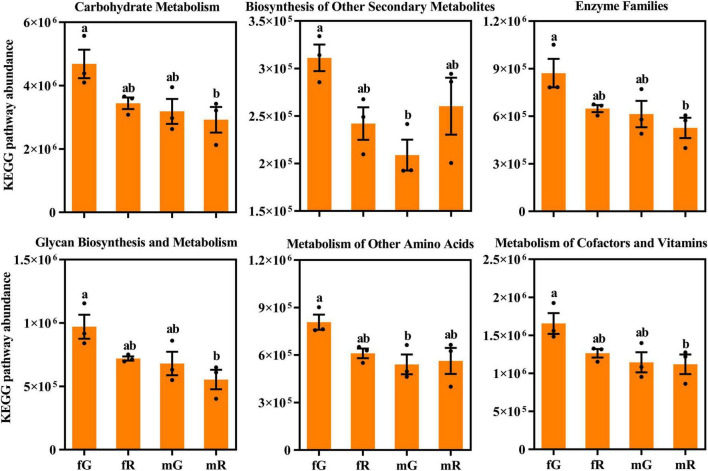
Comparisons of KEGG pathway absolute abundance among the fG, mG, fR, and mR of *B. dorsalis*. Values are means ± standard error (SE). Different letters (a or b) above the different column denote significant differences on KEGG pathway abundance.

Several differences in the absolute abundance of KEGG pathway related to the fG, mG, fR, and mR of *B. dorsalis* were revealed. Among the 6 pathways depicted in Figure 6, the absolute abundance of the pathway related to carbohydrate metabolism was highest (> 2.8 × 10^6^), while that related to biosynthesis of other secondary metabolites was lowest (< 3.1 × 10^5^). Among the fG, mG, fR, and mR of *B. dorsalis*, the abundances of pathways related to carbohydrate metabolism, enzyme families, glycan biosynthesis and metabolism, as well as metabolism of cofactors and vitamins were higher in fG compared to those in mR, and the abundances of pathways related to metabolism of other amino acids, and biosynthesis of other secondary metabolites were also higher in fG compared to those in mG (Figure 6).

## 4 Discussion

Different insect tissues harbor tissue-specific bacterial communities, as well as common or ubiquitous bacterial communities (Santos et al., 2023). Several studies have explored these bacterial communities in insect tissues, focusing on their richness and diversity (Jones et al., 2011; Chen et al., 2017; Kumar et al., 2023). The type and structure of bacteria present in insect tissues are closely linked to the complex physiological and biochemical characteristics of specific tissues (Collins and Patterson, 2020; Naveed et al., 2024). The study conducted here represents the most comprehensive exploration undertaken to-date of the overlapping and specific bacteria communities in the gut and reproductive system of *B. dorsalis* adults. Our results are instrumental in understanding the intrinsic relationship between the structure of bacterial communities and the function of tissues associated with these bacteria.

The removal of pair-end mismatches, homopolymers, and chimeric sequences ensured the quality of 16S rRNA gene sequence we obtained, which was beneficial for identifying the common and specific bacterial taxa and analyzing the functional pathway of bacteria enrichment. Meanwhile, over 30,000 clean reads per biological replicate were generated, providing sufficient RNA-seq data for analyzing the richness and diversity of bacterial taxa based on OTUs within *B. dorsalis* tissues. The higher numbers of clean reads and OTUs in fR and mR than in fG and mG (Table 1; Figure 1) indicated a greater diverse of bacterial taxa in the reproductive system of *B. dorsalis*.

Our study demonstrated that the richness indices of bacterial taxa in the reproductive system, particularly in the female reproductive system, were higher than those in the gut of *B. dorsalis* (Table 2). This finding is credible, because the environment of reproductive system is relatively stable, which provides more suitable living condition for its bacterial communities (Huang et al., 2010). Moreover, *B. dorsalis* adults were starved for 12 h before sampling, which eliminated a large number of bacteria via feces, particularly those retained in their guts (Andongma et al., 2015). The Venn diagram illustrated that the reproductive systems of *B. dorsalis* harbored a greater number of specific bacterial taxa compared to their guts. Our explanation is that the physiological and biochemical processes of the reproductive system are more diverse and complex, necessitating the participation of a greater variety of bacteria communities (Ben-Yosef et al., 2008). A study by Wang A. L. et al. (2014) also confirmed that the reproductive system of *B. minax*, a tephritid species closely related genetically to *B. dorsalis*, harbored more diverse and specific bacterial taxa than its gut.

We found that the bacterial community in different tissues of *B. dorsalis* was dominated by *Pseudomonadota*, followed by *Bacillota* and *Bacteroidota*. Other studies have also reported an abundance of *Pseudomonadota* in various insects, including *B. minax* (Wang A. L. et al., 2014), *Kerria lacca* (Kerr) (Kandasamy et al., 2022), and *Z. cucurbitae* (Lakshmi et al., 2023). On the contrary, bacterial communities associated with certain insects, such as *Apis mellifera* L. (Mohr and Tebbe, 2006) and *Coptotermes formosanus* Shiraki (Xiang et al., 2012), are more commonly dominated by *Bacillota* and *Bacteroidota*. Within the *Pseudomonadota*, members of the *Enterobacteriaceae* family constitute the majority of the bacterial community, which is also predominant in the guts of *C. capitata* (Marchini et al., 2002) and *Drosophila immigrans* (Berlin) (Jones et al., 2013). The wide distribution of Enterobacteriaceae suggested its important role in insect tissues. A study by Gu J. et al. (2024) revealed that some bacterial species within Enterobacteriaceae promoted the growth and development of *B. dorsalis* larvae through the vitamin B6 biosynthesis pathway. Furthermore, researches have shown that Enterobacteriaceae within insects may indirectly contribute to host adaptation, energy metabolism, and food digestion (Martinez et al., 2019; Wang et al., 2020; Zou et al., 2020).

The genera *Enterobacter*, *Kluyvera*, *Asticcacaulis*, *Mesorhizobium*, and *Serratia* were commonly found in the gut and reproductive system of *B. dorsalis*. Research has confirmed their widespread presence in the tissues of other insects, such as *C. capitata* (Behar et al., 2008), *P. papatasi* (Maleki-Ravasan et al., 2014), and *Bombyx mori* (Chen et al., 2018). These genera may play important roles in specific insect tissues. For instance, *Enterobacter* in the gut of *Helicoverpa armigera* (Hübner) is associated with the digestion and absorption of cellulosic food materials (Priya et al., 2012).

LEfSe analysis showed that the different tissues of *B. dorsalis* harbored their specific bacterial communities. Our study demonstrated that the gut of female *B. dorsalis* contained four specific bacterial taxa, namely, the class *Gammaproteobacteria*, the order *Enterobacteriales*, the family *Enterobacteriaceae*, and the genus *Morganella*. The guts of other fly species, such as *C. capitata* (Marchini et al., 2002) and *D. immigrans* (Jones et al., 2013) also harbored some members of Gammaproteobacteria. Furthermore, an increase in the abundance of Gammaproteobacteria in *S. gregaria* may enhance its immune defense against intestinal pathogens (Dillon et al., 2010). We also found that female adults of *B. dorsalis* harbored abundant members of the genus *Providencia*, whereas it had not been observed in male adults. A study by Roque-Romero et al. (2020) confirmed the presence of *Providencia* in female *Anastrepha obliqu*a (Macquart) as well. Furthermore, male *A. obliqua* that were fed with food containing *Providencia rettgeri* became more attractive to female adults of the same species, suggesting that *Providencia* may be associated with the courtship and mating behavior of female insects. Unfortunately, the roles of many tissue-specific bacterial communities in insects remain poorly understood. A possible reason is that the abundance of these bacteria in insect tissues is low and often overlooked.

The top 10 bacterial species, including *Enterobacter ludwigii*, *Enterococcus faecalis*, *Citrobacter freundii, Lactococcus lactis* and others, were identified by the BLAST algorithm. These species are common in many insect tissues, and their functions have also been revealed in some insect species (Priyadarsini et al., 2020). For example, Romero et al. (2006) demonstrated that *Citrobacter freundii* can stimulate oviposition in *Stomoxys calcitrans* L. (a fly species), and contribute to the development and reproduction of this fly species. Cheng et al. (2017) revealed that *C. freundii* may degrade trichlorphon (an organophosphate insecticide) into dimethyl phosphite and chloral hydrate, thereby enhancing the resistance of *B. dorsalis* to trichlorphon. Gwokyalya et al. (2023) found that *L. lactis* may improve the parasitic performance of parasitoids (*Fopius arisanus* and *Diachasmimorpha longicaudata*) against *B. dorsalis*, underscoring the critical role of bacterial symbionts in controlling this pest.

KEGG pathway analysis showed that the function of bacterial communities from the gut and reproductive system of *B. dorsalis* were mainly enriched in metabolism pathways, particularly those associated with carbohydrate metabolism, amino acid metabolism, and energy metabolism. This finding indicated that the majority of bacteria communities in the gut and the reproductive system of *B. dorsalis* are involved in the production, transformation, and utilization of food nutrients, thereby supporting their growth, development, and reproduction (Huang et al., 2017). Similar phenomena have also been demonstrated in other insect species, such as *Bombyx mori* L. and *Plutella xylostella* L. (Chen et al., 2017). By checking each of the 35 pathways annotated, we found that the abundances of the 2 pathways related to cardiovascular diseases and circulatory system were higher in the reproductive system compared to the gut. This phenomenon may be related to the reproduction of offspring in *B. dorsalis*. Among them, the function of the cardiovascular diseases pathway was likely to prevent genetically related disorders occurring in offspring, whereas that of the circulatory system pathway supported the production of robust offspring (Chen et al., 2024; Jama et al., 2024). The abundances of the 3 pathways associated with excretory system, membrane transport, and glycan biosynthesis and metabolism were all highest in the female gut (fG) of *B. dorsalis* among its four tissues. Clearly, the functions of these pathways are intricately tied to the absorption, utilization, and excretion of nutrients in *B. dorsalis* via its gut (Hu et al., 2011). Consequently, one plausible explanation for the high abundance in fG was that female *B. dorsalis* required a larger amount of food consumption to cope with more intense reproductive activity (Huang et al., 2017).

In summary, our research conducted here revealed the overlapping and specific bacterial communities between the gut and reproductive system of female and male *B. dorsalis*. Our study demonstrated that the reproductive system harbored more diverse and specific bacterial communities compared to the gut, whereas the female gut exhibited a higher abundance of function pathways than other tissues. Future studies should focus on revealing the roles of specific bacteria genera/species and identifying whether these bacteria could serve as targets for controlling *B. dorsalis*.

## 5 Conclusion

This study characterized the overlapping and specific bacterial communities in the gut and reproductive system of female and male *B. dorsalis*. Compared with guts, reproductive systems have more diverse and specific bacterial taxa based on OTUs. In bacterial communities, *Enterobacter*, *Kluyvera*, *Asticcacaulis* and *Mesorhizobium* were common, while *Gammaproteobacteria*, *Vagococcus*, *Providencia* and *Sphingobacteriia* exhibited tissue specificity. High-abundance bacterial communities were enriched in these function pathways of membrane transport, carbohydrate metabolism, amino acid metabolism, replication and repair, and energy metabolism. The abundance of pathway related to carbohydrate metabolism was highest in the gut of female adults, compared with other pathways. Our research demonstrated that the bacterial communities vary across different tissues of *B. dorsalis*, providing clues that elucidate the intrinsic relationship between these bacterial communities and the function of bacteria-associated tissues.

## Data Availability

The original contributions presented in the study are publicly available. This data can be found here: https://www.ncbi.nlm.nih.gov/, accession number: PRJNA1122372.

## Footnotes

1
http://www.ncbi.nlm.nih.gov/bioproject/

## Supplementary material

Supplementary Table S1 Information on the total OTUs within the gut and reproductive system of *B. dorsalis*.
